# Physicochemical Properties of Mixed Gelatin Gels with Soy and Whey Proteins

**DOI:** 10.3390/gels10020124

**Published:** 2024-02-03

**Authors:** Dong-Heon Song, Na-Eun Yang, Youn-Kyung Ham, Hyun-Wook Kim

**Affiliations:** 1 Department of Animal Science & Biotechnology, Gyeongsang National University, Jinju 52725, Republic of Korea; timesoul@naver.com; 2 Department of GreenBio Science, Gyeongsang National University, Jinju 52725, Republic of Korea; ly2223@naver.com; 3 Department of Animal Science, Sangji University, Wonju 26339, Republic of Korea; ykham21@sangji.ac.kr

**Keywords:** essential amino acid, hardness, jelly food, senior-friendly food

## Abstract

The physicochemical properties of the mixed gelatin gels with soy and whey proteins were investigated to develop the gel base with a soft texture and abundant essential amino acids for the elderly. Gelatin-only gel (control) was prepared at 6% (*w*/*v*), and mixed gelatin gels were formulated by replacing gelatin with soy protein isolate and whey protein concentrate at different mixing ratios [gelatin (G):soy protein isolate (S):whey protein concentrate (W)]. Results showed that replacing gelatin with the globular proteins in gelatin gels increased the pH value and processing yield (*p* < 0.05). Moreover, the mixed gelatin gels, particularly the G2:S1:W3 treatment, showed significantly higher essential amino acids than the gelatin-only control. The partial replacement of gelatin with the globular proteins could decrease the hardness of gelatin gel (*p* < 0.05), but there was no difference in hardness between the G2:G3:W1, G2:S2:W2, and G2:S1:W3 treatments (*p* > 0.05). The results of protein pattern, x-ray diffraction, and microstructure had no clear evidence for specific protein–protein interaction in the mixed gelatin gels. Therefore, this study indicates that mixed gelatin gels with the globular proteins at specific mixing ratios could be a practical approach to providing a soft texture and high-level essential amino acids to the elderly.

## 1. Introduction

In aging countries, texture-modified foods for the elderly, which belong to the category of senior-friendly foods, are designed to fulfill specific dietary conditions with physiological dysfunctions [1]. Older adults may have chewing, swallowing, and digestion problems due to dental issues, dysphagia, and dyspepsia [2]. Thus, the basic concepts for developing foods for the elderly generally involve texture modification with a soft structure and nutrient enhancement, mainly protein, to ameliorate age-related muscle loss and bone weakening [1,2]. Hydrocolloid gels formulated with proteins, polysaccharides, and other nutrients have been reasonably considered to provide a soft texture with high protein content for the elderly [1,3].

Gelatin, a protein derived from collagen, is commercially extracted from animal connective tissues in the skin, bone, and tendon of porcine and bovine [4]. Commercial pork or bovine gelatin is generally solubilized around 30–40 °C, whereas it reversibly forms a cold-set gel after cooling below the melting point [4]. Based on its rheological properties, gelatin is extensively used as a gelling agent in the food industry to produce jellies, gel desserts, puddings, mousse, and fruit fillings [5]. However, unfortunately, although gelatin is involved in an animal protein, it has a considerably lower digestible indispensable amino acid score than major grains (rice, wheat, oats, etc.) and pulses (soybeans and peas), particularly with tryptophan as the limiting amino acid and low levels of lysine and methionine [6]. Thus, it could be reasonable to incorporate other food protein ingredients rich in indispensable amino acids to improve nutritional value in developing texture-modified foods for the elderly.

Globular proteins extensively used in the food industry, such as egg-white protein, plant globulin, and whey proteins, have a gel-forming ability, mainly which produces irreversible heat-induced gels and aggregates through thermal denaturation [7]. Moreover, soy protein is a valuable protein source due to its availability and well-balanced nutritional value comparable to milk protein [8]. Whey protein is considered to have a high biological value due to high levels of indispensable amino acids and excellent digestibility [9]. Thus, incorporating such globular proteins could be an available way to enhance the limited amino acid composition in gelatin gels. However, there has been limited information on the combined inclusion of soy and whey proteins on the nutritional and gel properties of gelatin gel.

Protein–protein interactions in a binary protein gel system, including synergistic effects, phase separation, and aggregation, affect the overall rheological and textural properties of mixed protein gels [9]. Some previous studies have reported the physicochemical and rheological changes in gelatin gels mixed with other food proteins, mainly egg-white protein [10,11] and whey proteins [9,12]. Cai et al. [11] found that increasing egg-albumen protein levels (1%, 3%, and 5%) could increase the gel strength and storage modulus value of grass carp skin gelatin, resulting from synergistic protein–protein interactions. In addition, Sarbon et al. [9] reported that incorporating 3–5% gelatin with 10% whey protein isolate resulted in synergistic interaction increasing storage modulus. Thus, the gelatin gels mixed with other proteins could form a firm gel under specific concentrations.

As mentioned earlier, the primary objectives in developing protein gels for the elderly include easy chewing and swallowing due to their soft texture and a sufficient supply of nutrients. Although incorporating other proteins to supplement indispensable amino acids can effectively improve the nutritional value of gelatin gel, there is an apparent problem of increased firmness due to increased dry matter and synergistic protein–protein interactions [13]. Interestingly, a recent study evaluated whether adding two or more proteins to gelatin gel could suppress the increase in gel strength due to the synergistic effect between proteins [3]. In our previous study, adding soy protein and egg-white protein successfully produced a soft texture of the mixed gelatin gels despite the increased protein content [3]. It has been well-documented that whey protein has similar processing characteristics to egg-white protein and has been considered a commercial substitute for egg-white protein in various processed foods. In this regard, we chose whey protein to confirm the adding effect of globular protein in the mixed gelatin gel model. Taken together, it could be reasonable to hypothesize that incorporating multiple proteins rich in indispensable amino acids may be a practically effective processing strategy to produce gelatin-based nutritious and soft protein gel for the elderly. Therefore, this study was performed to evaluate the nutritional value, hardness, and specific protein–protein interaction of mixed gelatin gels with soy and whey proteins at various mixing ratios.

## 2. Results and Discussion

### 2.1. Processing Yield and pH

The processing yield and pH of the mixed gelatin gels are shown in Table 1. The processing yield of all treatments, ranging from 96.72% to 97.63%, was significantly higher than that of the gelatin-only control (G6:S0:W0, 95.82%). No difference (*p* > 0.05) in processing yield between mixed gelatin gel treatments was found. During thermos-reversible gelatin gelation, gelatin molecules undergo structural changes, forming a three-dimensional network that can immobilize water within the structure [4]. However, the water may not be expelled in the sense of being forced out. Previously, globular proteins, including soy protein isolate and whey protein concentrate, are well-known to have excellent water-binding and -holding capacities: 3.5–8.7 g water/g protein of soy protein isolate [14] and 1.9 g water/g protein of whey protein concentrates [15], respectively. However, according to Park and Kim [16], pig skin gelatin had a water absorption capacity below 12% (*w*/*w*). Thus, the increased processing yield of the mixed gelatin gels was probably due to the better water-binding capacity of the globular proteins added than gelatin.

The pH of the gelatin-only gel (control) was 4.87, which was a significantly lower pH than the mixed gelatin gels (6.36–6.81). This result could be related to the relatively acidic pH value of the pig gelatin powder (approximately 4.7). A similar result was previously found by Noh et al. [3], who noted that the inclusion of soy protein isolate (pH 7.30)/egg-white protein (pH 7.39) mixture increased the pH value of gelatin gels. Fundamentally, the gelation strength of gelatin is affected by the electronic environments surrounding the gelatin molecule, and the cross-linkage between gelatin molecules could be strengthened with predominant positive or negative charges at extreme acid and alkali pH, respectively [17]. Thus, the pH shifted into a neutral region due to the addition of globular proteins, which might be a chemical condition allowing the cross-linkage between gelatin molecules to be weakened.

### 2.2. Proximate Composition and Calorie Content

The ranges of proximate composition in the mixed gelatin gels were as follows (Table 2): moisture (92.81–93.67 g/100 g), protein (6.02–6.59 g/100 g), lipid (0.01–0.02 g/100 g), and ash (0.02–0.28 g/100 g). The mixing ratio between gelatin, soy protein isolate, and whey protein concentrate significantly affected the proximate composition of the mixed gelatin gels, except for lipid content (*p* > 0.05). However, numerically slight differences (below 1%) in the moisture, protein, and ash between gelatin-only gel (control) and the mixed gelatin gels were found. For protein content, all mixing ratios could successfully achieve the targeted protein content (6 g/100 g) in the mixed gels, which is a minimum requirement for senior-friendly foods in the Korean Industrial Standard [18]. Moreover, replacing pig gelatin with soy protein isolate and whey protein concentrate could increase the ash content of the mixed gelatin gels because commercial gelatin is generally demineralized [19]. The calorie content of the gelatin-only gel (control) was 26.16 kcal/100 g and replacing gelatin with the globular proteins could slightly increase the calorie content of the mixed gelatin gels (*p* < 0.05). Although it is essential for the elderly to consume sufficient calories and nutrients, excessive calorie intake can increase fat accumulation in the body due to reduced energy expenditure (reduced basal metabolic rate) compared to other age groups [20]. According to Gallego et al. [21], the need for texture-modified foods with a high density of calories and nutrients has been mentioned because older adults who consume senior-friendly foods consume less food. Thus, future research exploring the processing strategies to enhance the calories and nutrients (usually vitamins and minerals) in the mixed gelatin gels for the elderly should be studied without any adverse impacts on the quality attributes of final products.

### 2.3. Total Amino Acid

The total amino acid contents of the mixed gelatin gels are shown in Table 2, where seven essential amino acids and ten non-essential amino acids were detectable. Similar total amino acid content (4.73–4.89 g/100 g) was found (*p* < 0.05). However, the content of each free amino acid was significantly different, depending upon the mixing ratios. The subtotal of all detected essential amino acids was 0.84 g/100 g in the gelatin-only gel (control). Replacing gelatin with soy protein isolate and whey protein concentrate could significantly increase the content of the essential amino acids in the mixed gelatin gels. In particular, the highest content of the essential amino acids was found at G2:S2:W2 and G2:S1:W3 treatments (*p* < 0.05), which were prepared with relatively lower gelatin proportions than other treatments. In detail, as the proportion of whey protein concentrate at the same replacing ratio of gelatin increased (e.g., G3:S3:W0 vs. G3:S0:W3 or G2:S3:W1, G2:S2:W2, vs. G2:S1:W3), Lys, Leu, Thr, and Met in the mixed gelatin gels increased markedly. This result could be because whey protein concentrate contains relatively abundant amino acids. For non-essential amino acids, replacing gelatin with the globular proteins increased His, Glu, Asp, Ser, Tyr, and Cys but decreased Arg, Gly, and Ala.

Several common disorders and health issues commonly occur for the elderly, although the prevalence of specific diseases can vary. In particular, osteoporosis and sarcopenia are closely related to reduced protein intake and synthesis [22,23]. Previously, it has been reported that intake of specific amino acids is associated with the onset and worsening of disease symptoms through various mechanisms. Skeletal health is potentially related to the consumption of Arg and Lys (calcium absorption), His and Phe (osteoblast stimulation), and a combination of Phe, Try, and Trp (osteoclast inhibition) [22]. In skeletal muscle metabolism and function, branched-chain amino acids (e.g., Leu, Ile, and Val) predominantly play essential roles in the skeletal muscle [23]. Thus, supplementing gelatin with the globular proteins used in this study, particularly whey protein concentrate, could be a desirable approach to guarantee the nutritional value of gelatin-based foods for the elderly.

### 2.4. Hardness

**Figure 1:**
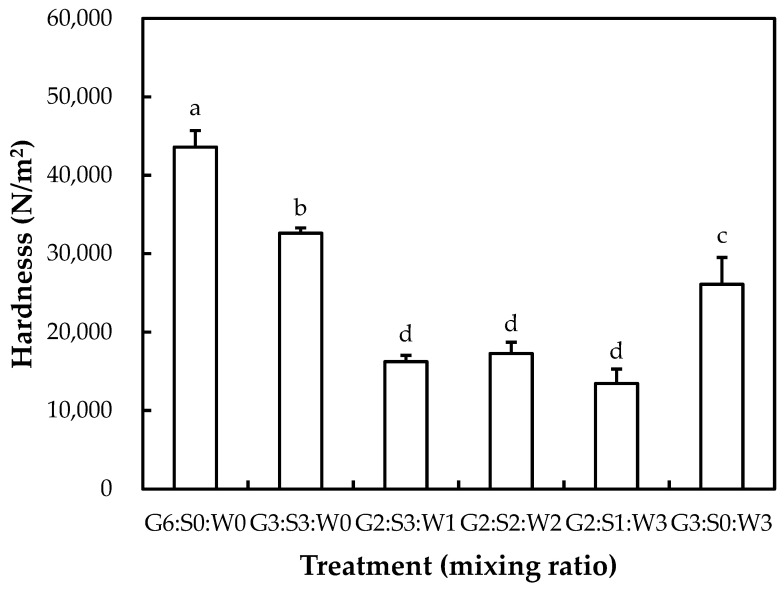
Hardness of the mixed gelatin gels with soy protein isolate and whey protein concentrate. G–pig skin gelatin; S–soy protein isolate; W–whey protein concentrate. Each bar refers to the standard deviation of the mean (*n* = 3). (a–d) Means with the same letters are not significantly different (*p* ≥ 0.05).

The hardness of the mixed gelatin gels is shown in Figure 1. The inclusion of the globular proteins at various ratios decreased the hardness of the gelatin gel (*p* < 0.05). As a result, the highest hardness (43,594 N/m^2^) was observed for gelatin-only gel (G6:S0:W0) (*p* < 0.05). The lowest hardness (13,439–17,261 N/m^2^) was observed at the mixing ratios of G2:G3:W1, G2:S2:W2, and G2:S1:W3 (*p* < 0.05). According to the senior-friendly foods in the Korean Industrial Standard [18], the hardness of senior-friendly foods is classified into three grades, based on the instrumental hardness and viscosity (only in the 3rd grade): 1st grade of tooth intake (50,000 < and ≤ 500,000 N/m^2^), 2nd grade of gum intake (>20,000 and ≤50,000 N/m^2^), and 3rd grade of tongue intake (<20,000 N/m^2^, >1500 mPa·s). In this study, G2:G3:W1, G2:S2:W2, and G2:S1:W3 treatments presented adequate hardness, included in the 3rd grade of the senior-friendly foods in the Korean Industrial Standard. Our results imply that partial replacement of gelatin with the globular proteins could be useful to produce a relatively soft gelatin gel base for older people while providing equivalent protein content and an abundance of essential amino acids.

Basically, the gelation of gelatin powder generally involves the serial process of hydration, dissolution, thermal-induced unfolding (if heated), and gelation due to cross-linkage formation below melting temperature during cooling, known as cold-set gelation [4]. Moreover, the gelation process is fundamentally affected by pH, concentration, temperature, ionic strength, and the presence of polymers such as proteins and polysaccharides [7]. Previously, the gelatin–globular protein interaction in mixed gelatin gel has been extensively studied; it has been documented that gelatin molecules could influence the stiffness of heat-induced globular protein gels during thermal treatment, whereas the formed microgels could further be associated with the strength of cold-set gelatin gel after cooling [24]. In this study, the changed proportion of gelatin might be the most critical factor related to the different hardness between the mixed gelatin gels. Similarly, McCann et al. [24] have found that soy–whey protein mixtures at different ratios (100/0, 70/30, 50/50, 30/70, and 0/100) could have the same gel strength, although the structural difference in the gels was observed. It was also consistent with our results since there was no difference (*p* > 0.05) in hardness between the G2:G3:W1, G2:S2:W2, and G2:S1:W3 treatments.

### 2.5. Protein SDS-PAGE

**Figure 2:**
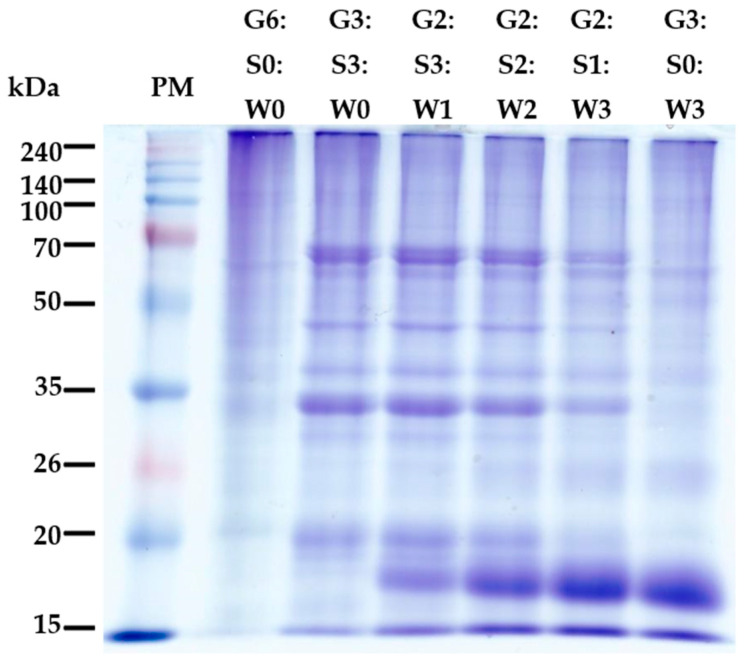
A representative photo of sodium dodecyl sulfate-polyacrylamide gel electrophoresis (SDS-PAGE) of the mixed gelatin gels with soy protein isolate and whey protein concentrate. PM–protein standard marker; G–pig skin gelatin; S–soy protein isolate; W–whey protein concentrate.

A representative photo of protein patterns of mixed gelatin gels is shown in Figure 2. Mammalian skin tissue is mainly composed of collagen type I, and previous studies have found that pig gelatin (type A) typically includes two α1 sub-chains and one α2 sub-chain derived from collagen type I, around the 100–140 kDa in the SDS-PAGE system [4]. In this study, the pig skin gelatin showed two distinct bands around 100 kDa, with several segment bands through the entire molecular weight below 100 kDa. Similar protein patterns of pig skin gelatin were observed previously, and the generation of segment bands could be related to the intensity of chemical pre-treatment and conditions for gelatin extraction [25,26]. As seen in the band patterns in G3:S3:W0 treatment, the inclusion of soy protein isolate resulted in the appearance of major soy protein bands, which are presumed as subunits of glycinin [an acidic polypeptide A (≈35 kDa) and a basic polypeptide B (≈20 kDa)] and β-conglycinin [α (≈72 kDa), α′ (≈68 kDa), and β (≈52 kDa)] [27,28]. In addition, as the proportion of whey protein concentrate increased, the intensity of soy protein bands declined, but that of newly generated bands due to adding whey protein concentrate, probably β-lactoglobulin (≈18 kDa), became clear. Previous studies on the interaction between gelatin and globular proteins have mainly observed the influence of globular protein aggregates on the cross-linking of gelatin molecules at the physical level, but chemical bonding between different protein molecules has not been observed through SDS-PAGE. Our results based on SDS-PAGE might also be insufficient to provide clear evidence for forming high molecular polymers through the molecular interaction between gelatin and the globular proteins used.

### 2.6. X-ray Diffraction (XRD)

**Figure 3:**
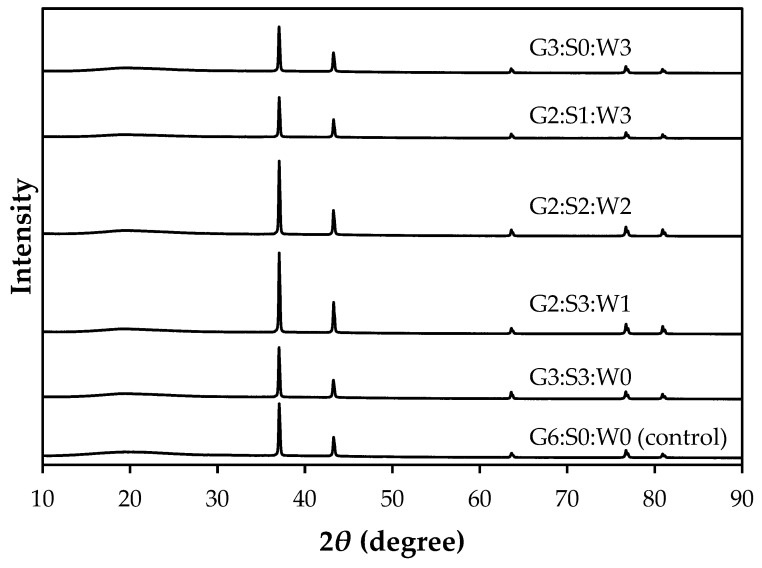
X-ray diffraction of the gelatin gels mixed with soy protein isolate and whey protein concentrate. G–pig skin gelatin; S–soy protein isolate; W–whey protein concentrate.

The XRD pattern of the mixed gelatin gels is shown in Figure 3. XRD is a useful technique used to investigate the structure of crystalline materials, including biological macromolecules like proteins [29]. In this study, the mixed gelatin gels showed similar XRD patterns with five distinct peaks at approximately 37°, 44°, 66°, 76°, and 81°. The double value of the diffraction angle, 2θ angle, is often used to show the position of diffraction peaks obtained from unique crystalline structures. The amorphous and crystalline intensity of samples was also similar between treatments, within 81.9–84.7% and 15.3–18.1%, respectively (*p* > 0.05, Table 3). Thus, the results for the XRD pattern in this study could indicate no significant change in the crystal lattice structure or spacing between crystal bases. While XRD is more commonly used for determining the three-dimensional structure of individual proteins, it can also be employed to gain insights into protein–protein interactions [30]. In this respect, our results can provide evidence that there were no remarkable changes in crystalline structure due to protein–protein interaction, but it may be insufficient to support differences in hardness between the mixed gelatin gels.

### 2.7. Microstructure

**Figure 4:**
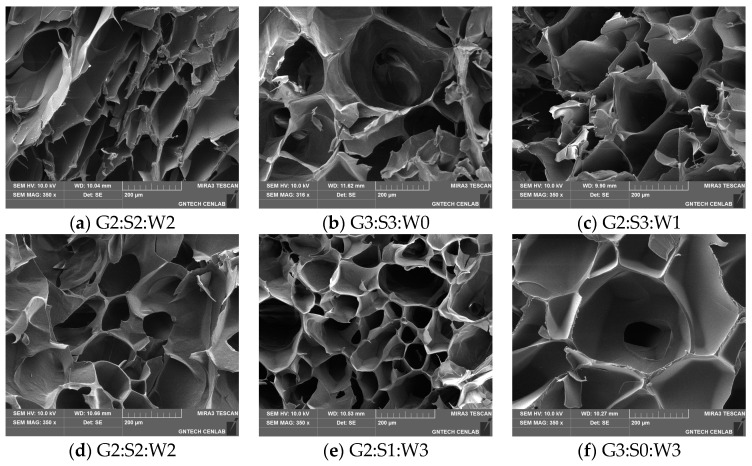
Microstructure of the mixed gelatin gels with soy protein isolate and whey protein concentrate observed by a scanning electron micrograph (SEM) at ×350. G–pig skin gelatin; S–soy protein isolate; W–whey protein concentrate.

The microstructure of the freeze-dried gelatin gels mixed with soy protein isolate and whey protein concentrate exhibited a spongy appearance due to the presence of different size pores (Figure 4), which was consistent with the results of XRD (Figure 3). Moreover, no visual characteristics on the arrangement and distribution of components entrapped in the gelatin gel were found, possibly presumed to be the microgels of globular proteins. Previously, Noh et al. [3] similarly observed the sponge-like structure of the freeze-dried gelatin gels mixed with soy protein isolate and egg-white protein and suggested that the morphological properties were likely due to the ice crystal formation. Thus, the stepwise heat-induced and cold-set gelation of the globular proteins and pig skin gelatin, respectively, might have little to no impact on the formation of unique microstructure in the mixed gelatin gels at the selected mixing ratios.

## 3. Conclusions

In conclusion, incorporating soy protein isolate and whey protein concentrate into gelatin gels at different mixing ratios significantly increased processing yield, likely attributed to the enhanced water-binding capacity of the added globular proteins. The pH shift towards a neutral region due to the inclusion of the globular proteins might be an electronic environment weakening gelatin cross-linkage. At different mixing ratios, proximate composition changes were minimal. Essential amino acid content improved in mixed gels, particularly the mixed gelatin gels with the increased proportion of whey protein concentrate, could be beneficial in mitigating issues like osteoporosis and sarcopenia in older adults. The partial replacement of gelatin with the globular proteins could decrease the hardness of gelatin gel, but there was no significant difference in hardness between different ratios of soy protein isolate and whey protein concentrate at the same gelatin level. To determine the specific protein–protein interaction, SDS-PAGE, XRD, and microstructural analysis revealed no significant changes in the crystalline structure, supporting the minimal impact on the unique microstructure of gelatin gels at the selected mixing ratios. Further research should explore processing strategies to establish the delicate design of senior-friendly foods based on mixed gelatin gels and investigate the sensory acceptance of the developed final products, guaranteeing the desirable texture modification and well-balanced calories and nutrients.

## 4. Materials and Methods

### 4.1. Raw Materials

The commercial products of pig gelatin (Italgel S.p.A., Cuneo, Italy, Bloom value of 200, protein content of 91.12 g/100 g), soy protein isolate (Sias, Cheongju, Republic of Korea, protein content of 76.65 g/100 g), and whey protein concentrate (Sewoo Inc., Gwangju, Republic of Korea, protein content of 77.02 g/100 g) were purchased from a local supplier [13].

### 4.2. Gel Preparation

As mentioned in our earlier paralleled study [3], the solid content using the protein powders was targeted at 6% (*w*/*v*). The protein powders were placed in a desiccator at room temperature for 12 h, and their solid contents were measured similarly (93.2–93.5 g/100 g). The mixed gelatin gels were formulated according to Table 4 [3], in which two replacement ratios (1:1 and 1:2) of gelatin with soy protein isolate and whey protein concentrate were considered. Although the protein content of the commercial food protein powders used in this study was slightly different, the protein powders were used with an equal amount to minimize changes in physicochemical properties due to differences in solid content. The weighed protein powders were dissolved in double distilled deionized water (DDDW) and homogenized at 11,000 rpm for 1 min using a homogenizer (Ultra-Turrax T25, IKA Labortechnik, Staufeni, Germany). Thirty milliliters of the protein solution (five aliquots per treatment) were dispensed into a polypropylene (PP) conical tube (50 mL) and sealed with a high-density polyethylene (HDPE) screw cap. The conical tubes were placed in a 90 °C water bath (JSIB-22T, JS Research Inc., Gongju, Republic of Korea) and heated for 30 min to induce the complete solubilization of the protein powders and further heat-induced gelation of soy protein isolate and whey protein concentrate. Before cooling, the heated samples were immediately re-homogenized to prevent layer separation and precipitation of denatured and cross-linked protein microgels. The mixed gelatin gel samples were sealed, placed in a refrigerator for further cold-set gelation of gelatin, and used for physicochemical analysis. Three independent batches were processed on different days (*n* = 3).

### 4.3. Physicochemical Analysis

#### 4.3.1. Processing Yield

The processing yield (%) of the mixed gelatin gels was determined according to the method of Gu et al. [13], based on the percentage weight difference after cold-set gelation

Processing yield (%) = [the weight of the sample before heating (g)/the weight of the sample after heating and cooling (g)] × 100

#### 4.3.2. pH

The pH of the mixed gelatin gels was measured using an electronic pH meter (Orion Star^TM^ A211 pH Benchtop Meter, Thermo Scientific Inc., Waltham, MA, USA) calibrated by the standard buffers (pH 4.0, 7.0, and 10.0) [3].

#### 4.3.3. Chemical Composition and Calorie Content

Moisture, protein, lipid, and ash contents of the mixed gelatin gels were analyzed by the Association of Official Analytical Chemists (AOAC) method [31], previously described by Noh et al. [3]. Caloric content was calculated based on the contents of proximate composition [32], and the equation was as follows:

Caloric content (kcal/100 g) = [protein content (g/100 g) × 4 kcal/g] + [lipid content (g/100 g) × 9 kcal/g]

#### 4.3.4. Total Amino Acid Profile

The total amino acid profile of the mixed gelatin gels was conducted according to the AOAC method [14], as described by Noh et al. [3]. The sample was hydrolyzed with 6 N HCl, filtered, air-dried, and dissolved with 0.02 N HCl. The sample was diluted with DDDW at 1:29 (*v*/*v*), and 20 μL of aliquots were injected into an amino acid analyzer (L-8900 Amino Acid Analyzer, Hitachi Ltd., Tokyo, Japan) equipped with Hitachi AAA PH column (#2622 PH column, 4.6 mm I.D. ×60 mm, Hitachi, Tokyo, Japan). The setting conditions of flow rate and temperature were 0.4 mL/min and 57 °C, respectively. The absorbance was read at 570 nm and 440 nm (for proline only). Each content of detected amino acids was expressed as g per 100 g.

#### 4.3.5. Hardness

The hardness of the mixed gelatin gels was determined according to the method for elderly foods in the Korean Industrial Standard [13,18]. Six hexagonal samples (1 cm in width, length, and height) were prepared from the core portions of the mixed gelatin gels. The hardness of the cubes was measured by a texture analyzer (CT3, Brookfield Engineering Laboratories, INC., Middleboro, MA, USA) equipped with a cylinder probe (10 mm in diameter, TA-10K). The measurement conditions for a twice 70% compression cycle test were as follows: 1 mm/s of pre-test speed, 2 mm/s of test speed, and 10 mm/s of post-test speed.

#### 4.3.6. Sodium Dodecyl Sulfate Polyacrylamide Gel Electrophoresis (SDS-PAGE)

The protein pattern of the mixed gelatin gels was analyzed using protein SDS-PAGE (12% separating and 4% stacking gels), according to the Laemmli method [33]. A standard protein marker (pre-stained DokDo-MARK, EBM-1032, Elpisbiotech) was used to determine the molecular weight of protein bands.

#### 4.3.7. X-ray Diffraction (XRD) Assay

The XRD assay was performed to evaluate the structural properties of the freeze-dried sample using an X-ray diffractometer (Ultima IV, Rigaku Co., Tokyo, Japan) with Cu-Kα radiation (40 kV/30 mA). The 10–90° (2*θ* value) in the sample was scanned at the speed of 2°/min [3]. The percentage intensity of amorphous and crystalline regions was calculated against total diffracted intensity [34].

#### 4.3.8. Microstructure

The microstructure of the freeze-dried sample was observed using a field emission scanning electron microscope (SEM, Mira3 LM Tescan, Brno, Czech Republic) at ×350 magnification. The sample preparation and observation conditions were performed according to the method of Noh et al. [3].

### 4.4. Statistical Analysis

Data were expressed as mean ± standard deviation. Statistical analysis of measured variables was conducted using one-way ANOVA of the SPSS 18.0 software package (SPSS Inc., Chicago, IL, USA). The significance between the means was determined by Duncan’s multiple range test (*p* < 0.05).

## Figures and Tables

**Table 1:** Processing yield, chemical composition, and calorie content of the mixed gelatin gels with soy protein isolate and whey protein concentrate.

Parameter	Mixing Ratio (Gelatin:Soy Protein Isolate:Whey Protein Concentrate)					
	G6:S0:W0 (Control) ^1^	G3:S3:W0	G2:S3:W1	G2:S2:W2	G2:S1:W3	G3:S0:W3
Processing yield (%)	95.82 ± 0.34 ^c^	96.72 ± 0.31 ^b^	97.04 ± 0.18 ^ab^	97.17 ± 0.40 ^ab^	97.28 ± 0.56 ^ab^	97.63 ± 0.15 ^a^
pH	4.87 ± 0.03 ^f^	6.52 ± 0.01 ^d^	6.81 ± 0.00 ^a^	6.75 ± 0.01 ^b^	6.71 ± 0.01 ^c^	6.36 ± 0.01 ^e^
Moisture (g/100 g)	93.67 ± 0.03 ^a^	93.31 ± 0.16 ^b^	92.90 ± 0.06 ^c^	92.88 ± 0.02 ^c^	92.81 ± 0.11 ^c^	92.85 ± 0.22 ^c^
Protein (g/100 g)	6.48 ± 0.05 ^ab^	6.02 ± 0.07 ^e^	6.25 ± 0.01 ^d^	6.29 ± 0.04 ^cd^	6.42 ± 0.07 ^bc^	6.59 ± 0.13 ^a^
Lipid (g/100 g)	0.01 ± 0.00	0.01 ± 0.01	0.02 ± 0.01	0.01 ± 0.00	0.01 ± 0.01	0.01 ± 0.00
Ash (g/100 g)	0.02 ± 0.00 ^e^	0.16 ± 0.00 ^c^	0.28 ± 0.05 ^a^	0.19 ± 0.01 ^bc^	0.21 ± 0.02 ^b^	0.11 ± 0.01 ^d^
Calorie (kcal/100 g)	26.16 ± 0.20 ^b^	26.23 ± 0.60 ^b^	27.40 ± 0.20 ^a^	27.80 ± 0.08 ^a^	28.05 ± 0.51 ^a^	28.28 ± 0.86 ^a^

^1^ G–pig skin gelatin; S–soy protein isolate; W–whey protein concentrate. a–f Means with the same letters within a row are not significantly different (*p* ≥ 0.05).

**Table 2:** Total amino acid content of the mixed gelatin gels with soy protein isolate and whey protein concentrate.

Parameter	Mixing Ratio (Gelatin:Soy Protein Isolate:Whey Protein Concentrate)					
	G6:S0:W0 (Control) ^1^	G3:S3:W0	G2:S3:W1	G2:S2:W2	G2:S1:W3	G3:S0:W3
Essential amino acids (EAA, g/100 g)						
Lys	0.21 ± 0.01 ^d^	0.24 ± 0.01 ^d^	0.31 ± 0.01 ^c^	0.33 ± 0.01 ^bc^	0.39 ± 0.02 ^a^	0.34 ± 0.02 ^b^
Leu	0.15 ± 0.01 ^e^	0.25 ± 0.01 ^d^	0.35 ± 0.02 ^c^	0.40 ± 0.02 ^b^	0.44 ± 0.03 ^a^	0.38 ± 0.01 ^bc^
Val	0.14 ± 0.01 ^c^	0.17 ± 0.00 ^b^	0.19 ± 0.01 ^a^	0.19 ± 0.00 ^a^	0.20 ± 0.01 ^a^	0.19 ± 0.00 ^a^
Thr	0.10 ± 0.01 ^e^	0.14 ± 0.00 ^d^	0.17 ± 0.01 ^c^	0.18 ± 0.01 ^ab^	0.19 ± 0.01 ^a^	0.17 ± 0.00 ^bc^
Phe	0.12 ± 0.01 ^b^	0.18 ± 0.01 ^a^	0.20 ± 0.01 ^a^	0.19 ± 0.00 ^a^	0.19 ± 0.03 ^a^	0.15 ± 0.01 ^b^
Ile	0.06 ± 0.00 ^d^	0.13 ± 0.00 ^c^	0.17 ± 0.01 ^ab^	0.19 ± 0.02 ^a^	0.18 ± 0.01 ^ab^	0.16 ± 0.01 ^b^
Met	0.05 ± 0.00 ^c^	0.06 ± 0.00 ^c^	0.07 ± 0.00 ^bc^	0.09 ± 0.02 ^a^	0.09 ± 0.00 ^a^	0.08 ± 0.00 ^ab^
Subtotal of EAA	0.84 ± 0.05 ^d^	1.16 ± 0.03 ^c^	1.45 ± 0.07 ^b^	1.57 ± 0.09 ^ab^	1.68 ± 0.10 ^a^	1.47 ± 0.05 ^b^
Non-essential amino acids (NEAA, g/100 g)						
His	0.05 ± 0.00 ^c^	0.08 ± 0.01 ^b^	0.10 ± 0.01 ^a^	0.10 ± 0.00 ^a^	0.10 ± 0.01 ^a^	0.08 ± 0.01 ^b^
Arg	0.45 ± 0.03 ^a^	0.39 ± 0.01 ^b^	0.35 ± 0.02 ^bc^	0.32 ± 0.01 ^c^	0.27 ± 0.01 ^d^	0.28 ± 0.01 ^d^
Gly	1.25 ± 0.09 ^a^	0.69 ± 0.00 ^b^	0.51 ± 0.03 ^c^	0.49 ± 0.00 ^c^	0.48 ± 0.01 ^c^	0.65 ± 0.01 ^b^
Pro	0.65 ± 0.05	0.67 ± 0.33	0.37 ± 0.02	0.42 ± 0.07	0.36 ± 0.01	0.42 ± 0.01
Ala	0.48 ± 0.03 ^a^	0.32 ± 0.01 ^bc^	0.29 ± 0.01 ^c^	0.29 ± 0.00 ^c^	0.30 ± 0.01 ^c^	0.35 ± 0.01 ^b^
Glu	0.54 ± 0.04 ^d^	0.70 ± 0.00 ^bc^	0.77 ± 0.04 ^a^	0.75 ± 0.00 ^ab^	0.73 ± 0.02 ^ab^	0.66 ± 0.02 ^c^
Asp	0.30 ± 0.02 ^c^	0.41 ± 0.00 ^b^	0.46 ± 0.02 ^a^	0.46 ± 0.01 ^a^	0.46 ± 0.01 ^a^	0.41 ± 0.01 ^b^
Ser	0.19 ± 0.01 ^c^	0.21 ± 0.00 ^b^	0.22 ± 0.01 ^a^	0.22 ± 0.00 ^a^	0.22 ± 0.01 ^a^	0.20 ± 0.00 ^ab^
Tyr	0.03 ± 0.00 ^c^	0.10 ± 0.00 ^b^	0.13 ± 0.01 ^a^	0.14 ± 0.01 ^a^	0.14 ± 0.03 ^a^	0.10 ± 0.01 ^b^
Cys	0.00 ± 0.01 ^e^	0.04 ± 0.00 ^d^	0.06 ± 0.00 ^c^	0.08 ± 0.00 ^b^	0.09 ± 0.00 ^a^	0.08 ± 0.00 ^b^
Total	4.81 ± 0.33	4.83 ± 0.41	4.79 ± 0.23	4.89 ± 0.20	4.89 ± 0.23	4.73 ± 0.14

^1^ G–pig skin gelatin; S–soy protein isolate, W–whey protein concentrate. a–e Means with the same letters within a row are not significantly different (*p* ≥ 0.05).

**Table 3:** Amorphous and crystalline intensity obtained from XRD patterns of the mixed gelatin gels with soy protein isolate and whey protein concentrate.

Parameter	Mixing Ratio (Gelatin:Soy Protein Isolate:Whey Protein Concentrate)					
	G6:S0:W0 (Control) ^1^	G3:S3:W0	G2:S3:W1	G2:S2:W2	G2:S1:W3	G3:S0:W3
Amorphous intensity (%)	81.9 ± 1.0	84.5 ± 3.4	83.8 ± 4.7	84.7 ± 3.5	84.6 ± 2.4	83.8 ± 2.1
Crystalline intensity (%)	18.1 ± 1.0	15.5 ± 3.4	16.2 ± 4.7	15.3 ± 3.5	15.4 ± 2.4	16.2 ± 2.1

^1^ G–pig skin gelatin; S–soy protein isolate; W–whey protein concentrate.

**Table 4:** Formula of the gelatin gels mixed with soy protein isolate and whey protein concentrate.

Ingredient	Mixing Ratio (Gelatin:Soy Protein Isolate:Whey Protein Concentrate)					
	G6:S0:W0 (Control) ^1^	G3:S3:W0	G2:S3:W1	G2:S2:W2	G2:S1:W3	G3:S0:W3
Pig skin gelatin (g)	6.0	3.0	2.0	2.0	2.0	3.0
Soy protein isolate (g)	-	3.0	3.0	2.0	1.0	-
Whey protein concentrate (g)	-	-	1.0	2.0	3.0	3.0
Double distilled deionized water (mL)	100.0	100.0	100.0	100.0	100.0	100.0

^1^ G–pig skin gelatin; S–soy protein isolate; W–whey protein concentrate. The basic concept of this table was cited from the previous study of Noh et al. [3].

## Data Availability

All data and materials are available on request from the corresponding author. The data are not publicly available due to ongoing research using part of the data.
